# miR-31 promotes oncogenesis in intrahepatic cholangiocarcinoma cells via the direct suppression of RASA1

**DOI:** 10.3892/etm.2013.1311

**Published:** 2013-09-18

**Authors:** CHENGHUAN HU, FEIZHOU HUANG, GANG DENG, WANPIN NIE, WEI HUANG, XI ZENG

**Affiliations:** Department of Hepatobiliary and Pancreatic Surgery, The Third Xiangya Hospital of Central South University, Changsha, Hunan 410013, P.R. China

**Keywords:** intrahepatic cholangiocarcinoma, microRNA-31, RAS p21 GTPase activating protein 1, proliferation, apoptosis

## Abstract

MicroRNAs (miRNAs) are involved in the pathogenesis of intrahepatic cholangiocarcinoma (ICC). However, the role of microRNA-31 (miR-31) in ICC has yet to be elucidated. In this study, we demonstrated that the expression of miR-31 was significantly upregulated in ICC tissues and the human ICC cell line HCCC-9810, when compared with that in normal adjacent tissues. Bioinformatic analysis and a dual-luciferase reporter assay revealed RAS p21 GTPase activating protein 1 (RASA1) to be a direct target of miR-31 in HCCC-9810 cells. Further investigation showed that the protein expression level of RASA1 was significantly decreased in ICC tissues, suggesting an inverse correlation between miR-31 and RASA1 expression during the tumorigenesis of ICC. Moreover, the forced downregulation of miR-31 by its inhibitor in HCCC-9810 cells significantly inhibited cell proliferation and promoted cell apoptosis. However, when the cells were cotransfected with miR-31 inhibitor and RASA1-specific small interfering RNA (siRNA), these changes were attenuated. Further analysis of the molecular mechanism showed that the activity of the RAS-mitogen-activated protein kinase (MAPK) signaling pathway was significantly decreased in miR-31-downregulated HCCC-8910 cells, while cotransfection with miR-31 inhibitor and RASA1-specific siRNA attenuated this effect. These results indicate that the downregulation of RASA1 by miR-31 promoted cellular proliferation and inhibited cellular apoptosis, partially by upregulating the activity of the RAS-MAPK signaling pathway in ICC. In conclusion, the present study revealed important regulatory functions of miR-31 and RASA1 in ICC, indicating that miR-31 and RASA1 may become promising diagnostic and/or therapeutic targets for ICC.

## Introduction

Intrahepatic cholangiocarcinoma (ICC) is a rare primary liver cancer originating from cholangiocytes. However, the disease is ranked as one of the top five causes of cancer-related mortality, and its five-year survival is poor, mainly due the fact that it is difficult to diagnose, as a result of its late clinical presentation. Furthermore, the disease has a high recurrence rate following surgical resection (1,2). Recent study of the molecular mechanism of ICC has shown potential for the development of a novel therapeutic strategy (3).

MicroRNAs (miRNAs), which are a type of endogenous non-coding RNA, are able to bind to the 3′ untranslated region (UTR) of their target mRNAs, leading to a block in translation or triggering the degradation of the target mRNAs (4). As a result, miRNAs act as endogenous agents of RNA interference. It has been demonstrated that miRNAs are crucial in the regulation of tumorigenesis (5). Several studies have shown that the expression of certain miRNAs is dysregulated in various types of cancer, and these miRNAs regulate a number of important oncogenes and anti-oncogenic genes at a post-transcriptional level, and are thus involved in the pathogenesis of cancer (6,7).

In ICC, a number of miRNAs, including microRNA-21 (miR-21), miR-31, miR-124 and miR-200c, have been demonstrated to show aberrant expression patterns (8–10), and these miRNAs may therefore participate in the tumorigenesis of this type of cancer. Several miRNAs have been revealed to be important in ICC (9,10). miR-200c was demonstrated to activate epithelial-mesenchymal transition (EMT) via directly targeting neural cell adhesion molecule 1 (NCAM1) in ICC (9). Furthermore, in hepatitis C virus (HCV)-related ICC, the low level of miR-124 expression mediated by the HCV core protein promoted ICC cell migration and invasion (10). However, the exact role of miR-31 in the development of ICC has yet to be elucidated.

RAS p21 GTPase activating protein 1 (RASA1) participates in the control of cellular proliferation and differentiation by enhancing the weak intrinsic GTPase activity of RAS proteins, thus producing the inactive GDP-bound form of RAS (11). A number of studies have shown that the dysregulation of RASA1 has an oncogenic effect in multiple types of cancer, including colorectal, liver and breast cancer, as well as promyelocytic leukemia (12–16). However, to date, it has not been elucidated whether RASA1 is important in the development of ICC, and the correlation between RASA1 and miR-31 in ICC cells has yet to be revealed.

The present study aimed to study the roles of miR-31 and RASA1 in the regulation of ICC cell proliferation and migration, in addition to elucidating the underlying molecular mechanisms. In conclusion, this study may be beneficial for the development of potential therapeutic targets for ICC.

## Materials and methods

### Materials and reagents

This study was approved by the Ethics Committee of the Third Xiangya Hospital (Changsha, China). Written informed consent was obtained from all participants. Thirty ICC samples and normal adjacent tissues were obtained from patients with ICC who underwent surgery at the Department of General Surgery of the Third Xiangya Hospital of Central South University.

The human HCCC-9810 cell line was purchased from the Institute of Biochemistry and Cell Biology at the Chinese Academy of Sciences (Shanghai, China). Dulbecco’s modified Eagle’s medium (DMEM) and fetal bovine serum (FBS) were obtained from Gibco (Grand Island, NY, USA). MTT was purchased from Sigma (St. Louis, MO, USA), while the TRIzol reagent, miRNA reverse transcription kit, psiCHECK™-2 vector and RASA1-specific small interfering RNA (siRNA) were obtained from Thermo Fisher Scientific (Waltham, MA, USA). The SYBR-Green qPCR Mix used in the study was purchased from Toyobo Corp. (Osaka, Japan) and the miR-31 inhibitor was purchased from Biomics Biotechnologies Co., Ltd. (Nantong, China). All the antibodies used in the western blotting were obtained from Abcam plc (Cambridge, UK) and the apoptosis detection kit was purchased from Merck Millipore (Darmstadt, Germany).

### Cell culture

The human HCCC-9810 cell line was cultured in DMEM containing 10% FBS and incubated at 37°C in a humidified incubator of 95% air and 5% CO_2_.

### Quantitative reverse transcription-polymerase chain reaction (qPCR) analysis

Total RNA was extracted using TRIzol reagent, in accordance with the manufacturer’s instructions. For the analysis of miR-31 expression, 2 *μ*g RNA was transcribed to cDNA using a stem-loop reverse transcription (RT) primer and an miRNA reverse transcription kit under the following conditions: 16°C for 30 min, 42°C for 30 min and 85°C for 5 min. The conditions for the PCR were as follows: 95°C for 5 min, 95°C for 20 sec and 60°C for 1 min, for 40 cycles. U6 gene was used as a normalization control. The relative quantity of miR-31 to U6 was calculated using the equation 2^−ΔCT^, in which ΔC_T_ = C_T,miR-31_ − C_T,U6_. For the mRNA expression, SYBR-Green qPCR mix was used in accordance with the manufacturer’s instructions. Specific primers in this study were synthesized by BGI (Guangzhou, China). The specific primers for RASA1 were as follows: sense, 5′-ACTTGACAGAACGATAGCAGAAG-3′ and antisense, 5′-GCCTCCGATCACTCTCTCTTA-3′. Human glyceralde-hyde-3-phosphate dehydrogenase (GAPDH) primers were used as a control (sense, 5′-GGCAGCCCAGAACATCATCC-3′ and antisense, 5′-GCCAGCCCAAGCATCAAAG-3′).

### Dual-luciferase reporter assays

A normal and a mutated 3′-UTR of RASA1 were constructed using PCR, and were then inserted into the multiple cloning sites in the psiCHECK-2 vector. For the luciferase assay, human HCCC-9810 cells were cultured to ~70–80% confluence in a six-well plate. The cells were subsequently cotransfected with psiCHECK-2-RASA1-3′-UTR or psiCHECK-2-mut RASA1-3′-UTR vector in combination with 100 nM miR-31 or 100 mM miR-31 inhibitor, respectively, using Lipofectamine 2000 (Invitrogen Life Technologies, Carlsbad, CA, USA). Following this, the cells were incubated with transfection reagent/DNA complex for 5 h, prior to being refreshed with fresh complete medium. A Dual-Luciferase^®^ Reporter assay system (Promega Corp., Madison, WI, USA) was used to assess the luciferase activities 48 h subsequent to the cotransfection. Renilla luciferase activity was normalized to firefly luciferase activity.

### Western blotting

Tissue samples were frozen solid using liquid nitrogen in a mortar and ground vigorously. Following this, the cell samples were rinsed twice with cold phosphate-buffered saline (PBS), prior to cold radioimmunoprecipitation assay (RIPA) buffer being used to lyse the protein from the tissue or cell samples. The concentration of protein was assessed using a bicinchoninic acid (BCA) assay kit (Thermo Fisher Scientific, Waltham, MA, USA). Subsequently, proteins (15 *μ*g per lane) were loaded onto 10% sodium dodecyl sulfate-polyacrylamide gel electrophoresis (SDS-PAGE) gel for separation, and were then electrophoretically transferred to polyvinylidene difluoride (PVDF) membranes. The proteins on the membranes were subsequently probed using primary antibodies, in accordance with the supplier’s instructions. The primary antibodies used included: mouse anti-human RASA1 monoclonal antibody, mouse anti-human GAPDH monoclonal antibody, rabbit anti-human Ras-related GTP-binding protein A polyclonal antibody (RAS-GTP), mouse anti-human RAS monoclonal antibody, rabbit anti-human pERK (phospho Y204) polyclonal antibody and mouse anti-human ERK1 monoclonal antibody. Following incubation with secondary antibodies (rabbit anti-mouse secondary antibody and mouse anti-rabbit secondary antibody), the results were visualized using peroxidase and an enhanced chemiluminescence system, and quantified with Quantity One^®^ software (Bio-Rad, Hercules, CA, USA).

### siRNA interference

Human HCCC-9810 cells were seeded at a density of 100,000 cells per well in six-well plates and cultured at 37°C in 5% CO_2_ for 24 h. The HCCC-9810 cells were then transfected with siRNA using Lipofectamine 2000, in accordance with the supplier’s instructions. Briefly, 100 nmol siRNA and 5 *μ*l Lipofectamine 2000 were diluted in opti-MEM^®^ (Invitrogen Life Technologies) to a final volume of 800 *μ*l. Following mixing for 20 min at room temperature, the siRNA/Lipofectamine 2000 mixture was added. The cells were subsequently incubated at 37°C in 5% CO_2_ for 6 h. Following incubation, the mixture was replaced with DMEM containing 10% FBS for 24 h.

### Proliferation assay

Cell proliferation was determined using an MTT assay. At 24 h post-transfection, the transfection medium in each well was replaced with DMEM containing 10% FBS, as used previously, and cultured for 12, 24, 36, 48 and 60 h. Subsequently, the medium was replaced with 100 *μ*l fresh serum-free medium and cultured with 0.5 g/l MTT. Following incubation at 37°C for 4 h, the MTT medium was removed by aspiration and 50 *μ*l dimethylsulfoxide (DMSO) was added to each well. The samples were then incubated at 37°C for a further 10 min, prior to the absorbance at 570 nm (A_570_) of each sample being measured using a plate reader (Multiskan MK3; Thermo Fisher Scientific).

### Apoptosis analysis

A flow cytometer (C6 Flow Cytometer System; Becton, Dickinson and Company. Franklin Lakes, NJ, USA) was used to assess the cell apoptosis with an Annexin V-fluorescein isothiocyanate (FITC) Apoptosis Detection kit (Merck Millipore). At 24 h post-transfection, the cells were harvested and washed twice with cold PBS, prior to 10^6^ cells being resuspended in 200 *μ*l binding buffer supplemented with 10 *μ*l Annexin-V-FITC and 5 *μ*l propidium iodide (PI)-phycoerythrin (PE). The cells were then incubated in the dark for 30 min. Following this, 300 *μ*l binding buffer was added and a flow cytometric analysis was performed.

### Statistical analysis

The results are expressed as the mean ± standard deviation of three independent experiments. The statistical analysis was performed using SPSS 19.0 statistical software (SPSS, Inc., Chicago, IL, USA). One-way analysis of variance (ANOVA) and the Student’s t-test were used to analyze all the data. P<0.05 was considered to indicate a statistically significant difference.

## Results

### Expression of miR-31 in ICC tissues and HCCC-9810 cells

**Figure 1.**
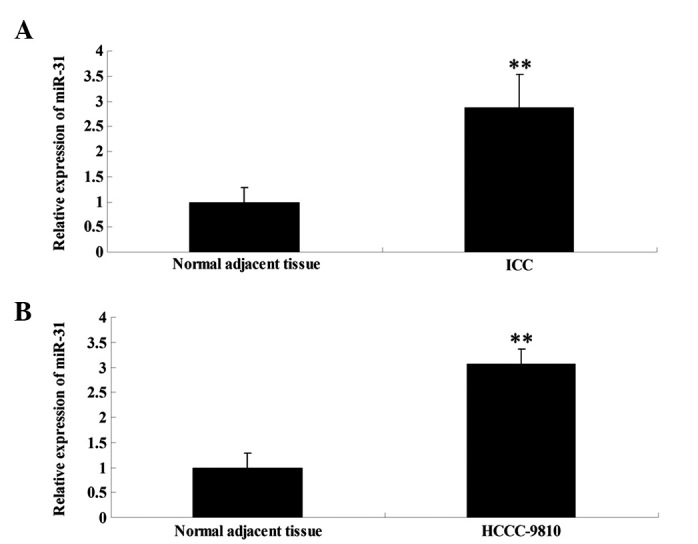
Expression of microRNA-31 (miR-31) in (A) intrahepatic cholangiocarcinoma (ICC) tissues and (B) the HCCC-9810 cell line. Quantitative reverse transcription-polymerase chain reaction was used to analyze the expression of miR-31 in 30 ICC samples. Normal adjacent tissues were used as controls. ^**^P<0.01 versus normal adjacent tissues.

To preliminarily investigate the role of miR-31 in ICC, we assessed the expression of miR-31 in ICC tissues and HCCC-9810 cells. Normal adjacent tissues were used as a control. As shown in Fig. 1A, the level of miR-31 expression in ICC tissue was significantly upregulated, when compared with that in normal adjacent tissues (P<0.01). We further examined the expression of miR-31 in the HCCC-9810 cell line. As shown in Fig. 1B, the level of miR-31 expression in HCCC-9810 cells was also significantly higher when compared with that in the control (P<0.01). These results suggest that miR-31 may be involved in the pathogenesis of ICC.

### RASA1 is a target of miR-31

To study the regulatory mechanism underlying the tumorigenesis of ICC, we investigated the targets of miR-31. Six types of commonly used bioinformatic software, including miRanda (17), miRDB, miRWalk (18), RNAhybrid (19), PICTAR5 (20) and Targetscan (21), independently predicted that RASA1 was a direct target of miR-31. Since these six types of software were based on different algorithms, the false positive rate of this predication was very low.

**Figure 2.**
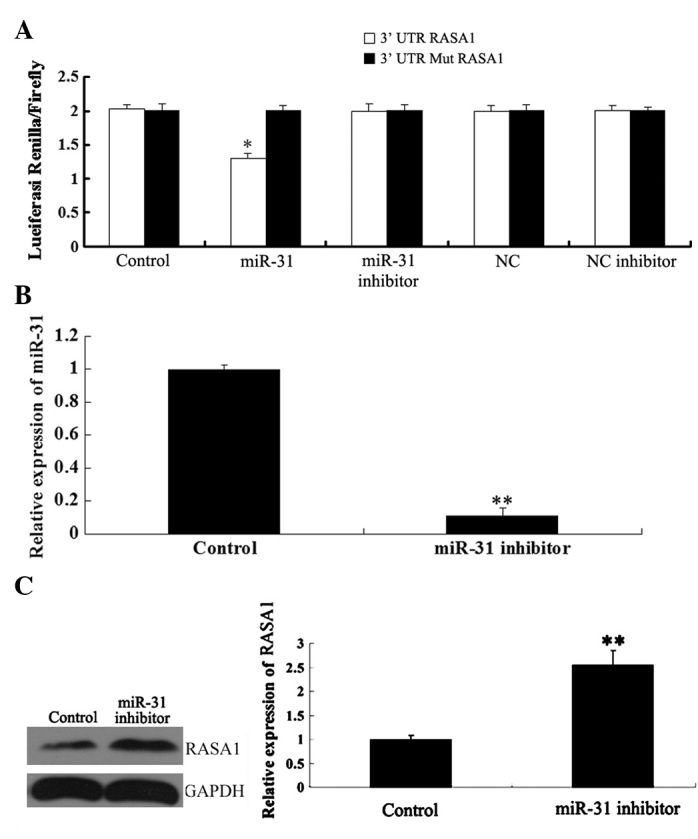
RAS p21 GTPase activating protein 1 (RASA1) is negatively regulated by microRNA-31 (miR-31). (A) Luciferase assay was used to assess whether RASA1 was the direct target of miR-31. A normal and a mutated 3′ untranslational region (UTR) of RASA1 were subcloned into the psiCHECK™-2 luciferase miRNA expression reporter vector. PsiCHECK™-2-RASA1-3′-UTR or psiCHECK™-2-mut RASA1-3′-UTR vector plus 50 nM miR-31 or 100mM miR-31 inhibitor were cotransfected into HCCC-9810 cells. ^*^P<0.05 versus control. (B) Quantitative reverse transcription-polymerase chain reaction was used to assess the expression of miR-31 in HCCC-9810 cells transfected with miR-31 inhibitor. ^**^P<0.01 versus control. (C) Western blotting was used to examine the protein expression of RASA1 in HCCC-9810 cells transfected with miR-31 inhibitor. ^**^P<0.01 versus control. Control, HCCC-9810 cells; NC, HCCC-9810 cells transfected with NC virus; NC inhibitor, HCCC-9810 cells transfected with NC inhibitor. GAPDH, glyceraldehyde-3-phosphate dehydrogenase.

Based on the results of the bioinformatic analysis, we performed a dual-luciferase reporter assay to investigate whether RASA1 was a direct target of miR-31. In miR-31 and RASA1-3′-UTR-cotransfected HCCC-9810 cells, the renilla/firefly value of luciferase was notably decreased (P<0.05). However, in the miR-31 and RASA1 mutated 3′-UTR-cotransfected HCCC-9810 cells, the renilla/firefly value of luciferase showed no difference from that in the control cells (Fig. 2A). These data identified RASA1 as a direct target of miR-31.

To further confirm these results, we transfected miR-31 inhibitor into HCCC-9810 cells and then examined the expression of miR-31, as well as the protein level of RASA1. Following transfection with miR-31 inhibitor, the expression level of miR-31 was significantly reduced (P<0.01; Fig. 2B), while the protein expression of RASA1 showed an increase (P<0.01; Fig. 2C). This indicated that the RASA1 was a direct target of miR-31 in HCCC-9810 cells.

### mRNA and protein expression of RASA1 in ICC tissues

**Figure 3.**
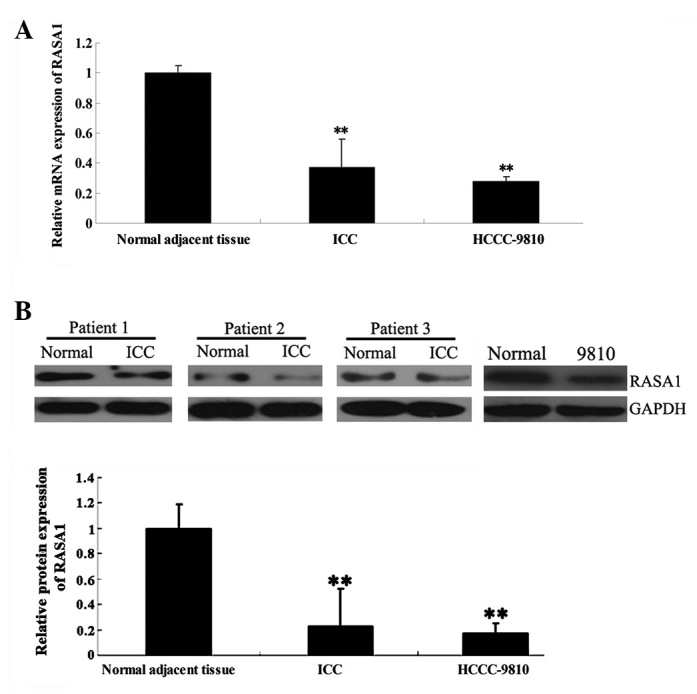
mRNA and protein expression of RAS p21 GTPase activating protein 1 (RASA1) in intrahepatic cholangiocarcinoma (ICC) tissues and HCCC-9810 cells. (A) Quantitative reverse transcription-polymerase chain reaction was used to assess the mRNA levels of RASA1 in 30 ICC samples and HCCC-9810 cells. Normal adjacent tissues were used as a control. ^**^P<0.01 versus control. (B) Western blotting was performed to examine the protein expression of RASA1 in 30 ICC samples and HCCC-9810 cells. Normal adjacent tissues were used as control. Glyceraldehyde-3-phosphate dehydrogenase (GAPDH) was used as an internal reference. Three typical results from patients with ICC are shown. ^**^P<0.01 versus control.

Based on the previously mentioned results, we further examined the mRNA and protein expression of RASA1 in the ICC samples and normal adjacent tissues, as well as in HCCC-9810 cells. As demonstrated in Fig. 3A, the mRNA expression level of RASA1 was markedly downregulated in ICC tissues and HCCC-9810 cells when compared with that in normal adjacent tissues (P<0.01). As shown in Fig. 3B, the protein expression of RASA1 was also significantly downregulated in ICC tissues and HCCC-9810 cells when compared with that in normal adjacent tissues (P<0.01). These data suggest an inverse correlation between miR-31 and RASA1 expression during the tumorigenesis of ICC.

### Roles of miR-31 and RASA1 in HCCC-9810 cell proliferation

**Figure 4.**
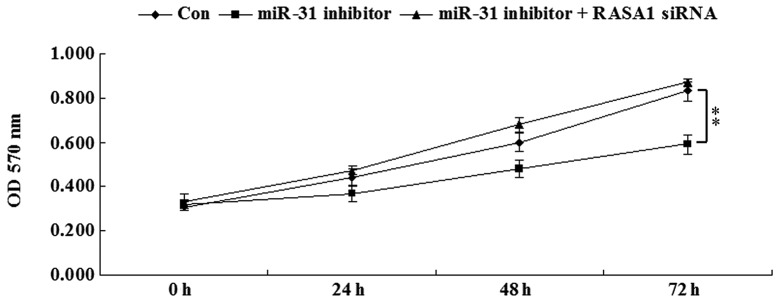
Roles of microRNA-31 (miR-31) and RAS p21 GTPase activating protein 1 (RASA1) in the regulation of HCCC-9810 cell proliferation. An MTT assay was performed to analyze the roles of miR-31 and RASA1 in the proliferation of HCCC-9810 cells. Con, normal HCCC-9810 cells without any treatment; miR-31 inhibitor, HCCC-9810 cells transfected with miR-31 inhibitor; miR-31 inhibitor + RASA1 siRNA, HCCC-9810 cells cotransfected with miR-31 inhibitor and RASA1 small interfering RNA. ^**^P<0.01 versus control. OD, optical density.

To further study the effects of miR-31 and RASA1 on the proliferation of HCCC-9810 cells, an MTT assay was performed. As shown in Fig. 4, in miR-31-downregulated HCCC-9810 cells, the cell proliferation rate was significantly decreased when compared with that in the control untreated HCCC-9810 cells (P<0.01), indicating that miR-31 was able to enhance cell proliferation. However, in HCCC-9810 cells cotransfected with miR-31 inhibitor and RASA1 siRNA, the cell proliferation rate was not downregulated, and instead showed a marginal increase when compared with that in the control group, further indicating that RASA1 acted as a downstream effector of miR-31 and was important in the regulation of ICC cell proliferation. Moreover, these observations also suggested that the ability of miR-31 to accelerate cell proliferation was partially via the direct suppression of RASA1 expression.

### Roles of miR-31 and RASA1 in HCCC-9810 cell apoptosis

**Figure 5.**
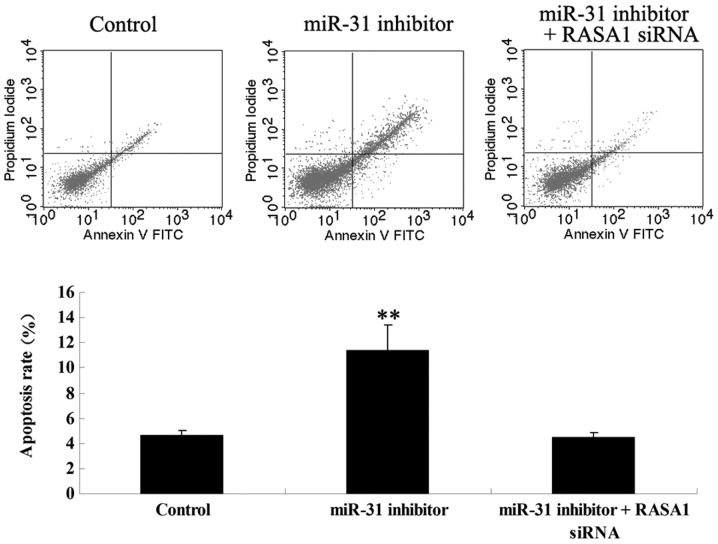
Effects of microRNA-31 (miR-31) and RAS p21 GTPase activating protein 1 (RASA1) in HCCC-9810 cell apoptosis. An Annexin V-FITC apoptosis detection kit was used to assess the cell apoptosis of HCCC-9810 cells in each group using flow cytometry. Con, normal HCCC-9810 cells without any treatment; miR-31 inhibitor, HCCC-9810 cells transfected with miR-31 inhibitor; miR-31 inhibitor + RASA1 siRNA, HCCC-9810 cells cotransfected with miR-31 inhibitor and RASA1 small interfering RNA. ^**^P<0.01 versus control.

To investigate the roles of miR-31 and RASA1 in HCCC-9810 cell apoptosis, Annexin V/PI double-staining and flow cytometric analysis were performed. As shown in Fig. 5, in miR-31-downregulated HCCC-9810 cells, the cell apoptosis rate was significantly higher than that in untreated HCCC-9810 cells (P<0.01), suggesting that miR-31 negatively regulated cell apoptosis in ICC cells *in vitro*. However, in HCCC-9810 cells cotransfected with miR-31 inhibitor and RASA1 siRNA, the cellular apoptosis rate was not increased compared with that of untreated HCCC-9810 cells, suggesting that miR-31 was able to inhibit cell apoptosis in HCCC-9810 cells, possibly by downregulating RASA1.

### Molecular mechanism for miR-31 and RASA1 in ICC

**Figure 6.**
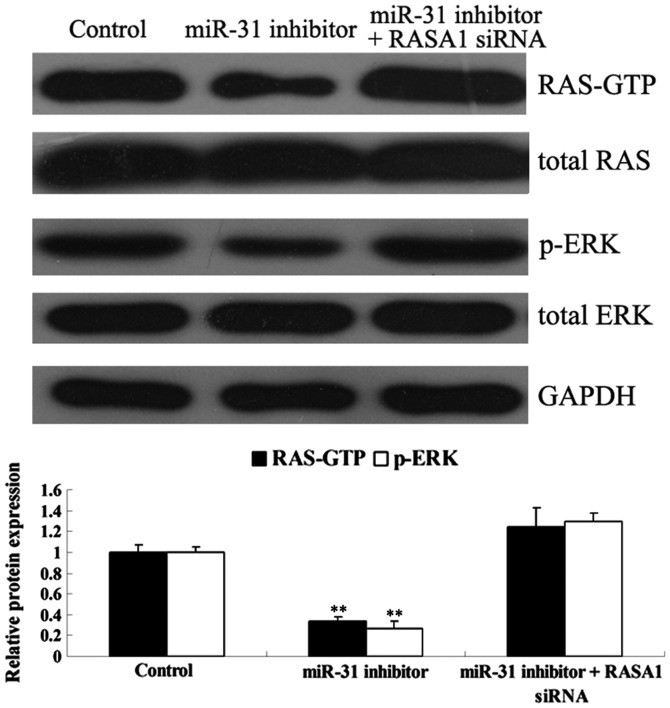
Roles of microRNA-31 (miR-31) and RAS p21 GTPase activating protein 1 (RASA1) in the regulation of the mitogen-activated protein kinase (MAPK) signaling pathway in HCCC-9810 cells. The regulatory roles of miR-31 and RASA1 were investigated using western blotting. Glyceraldehyde-3-phosphate dehydrogenase (GAPDH) was used as an internal reference. RAS-GTP, GTP-bound RAS; p-ERK, phosphorylation level of extracellular signal-regulated kinase 1/2; Control, normal HCCC-9810 cells without any treatment; miR-31 inhibitor, HCCC-9810 cells transfected with miR-31 inhibitor; miR-31 inhibitor + RASA1 siRNA, HCCC-9810 cells cotransfected with miR-31 inhibitor and RASA1 small interfering RNA. ^**^P<0.01 versus control.

It has been shown that RASA1 acts as a suppressor of the activity of the RAS-MAPK signaling pathway, which is crucial in the regulation of cell proliferation and apoptosis in various types of cancer (22,23). Therefore, we assessed the protein level of the GTP-bound RAS and the phosphorylation level of extra-cellular signal-regulated kinase 1/2 (ERK1/2), both of which are characteristic of the RAS-MAPK signaling pathway. As demonstrated in Fig. 6, in HCCC-9810 cells transfected with miR-31 inhibitor, the protein level of the GTP-bound RAS was significantly reduced when compared with that in the control group (P<0.01); however, cotransfection with miR-31 inhibitor and RASA1-specific siRNA attenuated this change. Moreover, in HCCC-9810 cells transfected with miR-31 inhibitor, the ratio of phospho-ERK1/2 to total ERK1/2 was markedly decreased, while cotransfection with miR-31 inhibitor and RASA1-specific siRNA also attenuated this change. These results partially explain why the forced downregulation of miR-31 inhibited cell proliferation and promoted cell apoptosis in ICC cells.

## Discussion

Previous studies have suggested that a number of miRNAs participate in the pathogenesis of ICC, including miR-21, miR-122, miR-145, miR-146a and miR-204 (8,10,24,25). In the present study, we showed that the expression of miR-31 was upregulated in ICC tissues. miR-31 has been demonstrated to be important in various types of cancer, including hepatocellular, squamous cell, ovarian, prostate and urothelial carcinoma, as well as colon, head and neck, gastric and breast cancer (8,26–33). However, the role of miR-31 in ICC has yet to be elucidated. As a result, we further investigated its regulatory mechanism in ICC cells.

Since miRNAs are generally involved in the pathogenesis of cancer by directly regulating the expression of their targets at a post-transcriptional level, we applied bioinformatic methods to predict the potential targets of miR-31. Our data revealed RASA1 to be a direct target of miR-31. More importantly, the protein level of RASA1 was increased in ICC tissues when compared with that in normal samples. Based on the contrasting expression patterns of miR-31 and RASA1, we proposed that miR-31 was involved in the pathogenesis of ICC by directly inhibiting the protein expression of RASA1, a validated oncogene in colorectal, liver and breast cancer, as well as promyelocytic leukemia (12–16). To the best of our knowledge, no previous investigations of this nature have been performed previously for ICC.

In order to assess the previously mentioned speculation, further investigation was performed. It was observed that the forced downregulation of miR-31 significantly promoted the protein expression of RASA1, inhibited ICC cell proliferation and enhanced cell apoptosis; however, the forced downregulation of RASA1 expression attenuated these changes, suggesting that RASA1 acted as a downstream effector of miR-31 in ICC cells.

It has been demonstrated that RASA1 stimulates the GTPase activity of normal RAS p21 and that the aberrant downregulation of RASA1 leads to abnormal cellular growth and proliferation, via the downstream RAS-MAPK signaling pathway, which has anti-apoptotic and pro-survival effects in numerous types of cancer (22,34). Based on this, we further investigated the activity of the RAS-MAPK signaling pathway in each group by examining the protein level of GTP-bound RAS, as well as the phosphorylation level of ERK1/2. Consistent with the previously mentioned studies, we showed that the forced downregulation of miR-31 significantly inhibited the activity of the RAS-MAPK signaling pathway, possibly through the upregulation of RASA1 expression; however, the downregulation of RASA1 attenuated this change. These data suggest that miR-31 promotes GTP binding to RAS by suppressing the expression of RASA1, and further upregulates the activity of the RAS-MAPK signaling pathway by increasing the phosphorylation level of ERK1/2 in ICC cells. Moreover, these results partially explain why the downregulation of miR-31 inhibits the cellular proliferation and promotes the apoptosis of ICC cells.

In conclusion, the present study provides a novel insight into the regulatory pattern of miRNA-31 and RASA1 in ICC *in vitro*, suggesting that miRNA-31 and RASA1 may become promising candidates for the development of effective strategies for the treatment of ICC.
